# Rate of cesarean delivery among patients with class III obesity at term based on body mass index: A retrospective cohort study

**DOI:** 10.1002/ijgo.71060

**Published:** 2026-05-11

**Authors:** Emily R. Root, Brittany A. Myers, Jeremy T. Gaskins, Anish Mukherjee, Rebecca C. Pierson, Edward E. Miller, Jessica Kline

**Affiliations:** ^1^ Department of Obstetrics and Gynecology Baylor College of Medicine Houston TX USA; ^2^ Department of Obstetrics, Gynecology and Women's Health University of Louisville Louisville Kentucky USA; ^3^ Department of Bioinformatics and Biostatistics University of Louisville Louisville Kentucky USA; ^4^ Division of Maternal Fetal Medicine, Department of Obstetrics, Gynecology and Women's Health University of Louisville Louisville Kentucky USA

**Keywords:** cesarean section, induction of labor, obesity, pregnancy

## Abstract

**Objectives:**

This study investigates the risk of cesarean delivery based on body mass index (BMI) in patients with class III obesity who undergo induction of labor.

**Method:**

This was a retrospective cohort study set in a university hospital. Study participants included patients presenting for delivery at term with class III obesity at the time of admission. A retrospective chart review was conducted of patients with a pre‐delivery BMI ≥40 who delivered between May 2016 and December 2020 at a single institution. Patients were divided into the following BMI groups: 40–44.9, 45–49.99, 50–59.99, ≥60. Demographic and other clinical features were analyzed using analysis of variance and Fisher tests across BMI groups. Rates of labor induction and cesarean delivery (CD) were further analyzed by logistic regression.

**Results:**

We identified 1010 patients with pre‐pregnancy BMI ≥40 who delivered in the study period, of whom 497 met the study criteria. The median BMI in this population was 43.9 (interquartile range: 41.8–48.5, range 40.0–92.9). Twenty‐seven percent of patients who underwent an induction of labor (IOL) delivered via CD compared to 12% for patients who presented in spontaneous labor. There were higher rates of induction seen in patients as BMI increased, but this effect was not seen in patients with BMI 60 or higher. The rate of CD was increased in patients who underwent IOL (*P* = 0.0332). This finding was only seen for those undergoing an elective IOL (*P* = 0.0088) but not a medically indicated IOL (*P* = 0.3588).

**Conclusion:**

Rates of induction of labor are higher in patients with higher BMI. In our cohort, those who underwent elective IOL were more likely to deliver via CD. Further study is needed to evaluate outcomes for patients with a BMI of 60 and greater, and more clarity is needed around recommendations for timing of elective induction of labor in patients with class III obesity.

## 1 INTRODUCTION

According to the National Health and Nutrition Examination Survey, 41.3% of women in the United States have obesity, and 12.1% of women meet criteria for severe obesity, or class III obesity (BMI ≥40).^1^ Obesity in pregnancy is associated with increased risks during the antepartum, intrapartum, intraoperative, postoperative, and postpartum periods.^2^, ^3^ Obesity in pregnancy has further been shown to be associated with prolonged gestation and subsequent late‐term delivery, which can increase the likelihood that patients with obesity will undergo induction of labor (IOL).^2^, ^3^ Recent studies have shown that inductions of labor after 39 weeks do not increase cesarean delivery (CD) rates.^4^ However, patients with class III obesity are not represented in these major trials, creating a gap in our knowledge. Patients with class III obesity are more likely to have severe maternal morbidity (SMM), postpartum hemorrhage, venous thromboembolism, and surgical site infections.^3^ It is important as providers to take particular care to reduce the incidence of CD in those with class III obesity to prevent SMM and its sequelae.

A recent systematic review and meta‐analysis demonstrate that few studies evaluate outcomes for patients with BMI ≥40 who undergo IOL.^3^ Patients with obesity are often evaluated in a single group based their diagnosed obesity class, but there are few studies that have evaluated outcomes within sub‐groups of these classes.^5^ A systematic review and meta‐analysis performed by D'Souza et al. determined that there was a dose‐dependent relationship between increasing BMI class and risk of CD, although this study included papers that did not evaluate the subgroups within each class.^6^ There is a growing body of literature reporting the outcomes regarding mode of delivery for those with class III obesity, but the findings of these studies are mixed, with some reporting increasing rates of CD with increasing BMI.^6^, ^7^, ^8^, ^9^, ^10^, ^11^, ^12^, ^13^, ^14^ Few studies evaluate the risk of CS in patients undergoing labor induction versus spontaneous labor by BMI category among those with class III obesity.^15^

To better understand how increasing BMI affects the rate of CD, studies that stratify patients with class III obesity into subgroups are needed to provide comprehensive personalized care. This study aims to describe outcomes at our institution for women with class III obesity who underwent IOL based on BMI subgroup.

## 2 METHODS

This was a retrospective cohort study of singleton deliveries to patients with class III obesity conducted at a single tertiary care, level IV maternity hospital in Louisville, Kentucky, USA, between May 1, 2016, and December 31, 2020.

### 2.1 Patient selection

All singleton deliveries ≥37 weeks were screened for eligibility. Patients were included if they delivered at our university hospital, were admitted for either IOL or were in spontaneous labor, with class III obesity documented (BMI ≥40, based on a calculation of the patient's body weight on admission and the patient's height), and complete documentation within the chart. Patients were excluded if they had a history of cesarean section and were presenting for trial of labor, had been admitted for a planned cesarean section, had a BMI <40, or had incomplete records. For patients with multiple deliveries within the study period, the first delivery was included in analyses, and subsequent deliveries were excluded.

### 2.2 Data collection

A retrospective chart review was performed from the electronic medical record using standardized forms. The data collected included: maternal demographics, comorbidities, labor course, delivery outcomes, and neonatal data (further details are listed in Appendix S1). Data was extracted from the medical record based on diagnoses included in patients' charts. The patients' labor course was reviewed in the provider documentation of the labor course. Data was abstracted by two trained reviewers using standardized forms with 10% double‐abstraction (*κ* > 0.85). Complete case analysis was defined as less than 5% missing primary outcome.

### 2.3 Statistical analysis

Patients were categorized into four BMI groups: 40.0–44.9, 45.0–49.9, 50.0–59.9, and 60.0+. There are limited classification systems for stratifying patients with class III obesity. Some categorization systems stratify those with a BMI of 40–49.99 as severe obesity and super obesity defined as BMI greater than 50. Studies that evaluate health outcomes in different disciplines have used four categories for patients with class III obesity: BMI 40.0–44.9, 45.0–49.9, 50.0–59.9, and 60.0+ schema.^16^ Given the differences in health outcomes noted for patients by BMI group in the more granular approach in other studies, we adopted this method to evaluate our cohort.

Demographic and other clinical features were compared across BMI groups using analysis of variance for continuous features and Fisher tests for binary/categorical features. Logistic regression was used to consider the impact of BMI group on the primary outcomes of IOL and cesarean delivery, while controlling for maternal age, null parity, and chronic diseases such as hypertensive disorders in pregnancy (HDP) and diabetes. The model for CD also included IOL as a predictor. A significance level of *α* = 0.05 was used throughout and all statistical analysis was performed using R statistical software, version 4.1.2.

#### 2.3.1 Ethics

The study was approved by the University of Louisville Institutional Review Board. Informed consent was waived due to the retrospective nature of the study.

## 3 RESULTS

### 3.1 Study population

Within the study period, 1010 deliveries were identified during the study period in patients with a BMI of 40 or greater. 513 deliveries were excluded due to scheduled or repeat CD, vaginal birth after CD, multiple gestation, intrauterine fetal demise, or having more than one delivery within the study period. A total of 497 deliveries met the study criteria and were included in the analysis. The average age (± standard deviation) of included patients was 27.5 ± 6.1 years. Approximately one‐third (35.6%) of patients in the study were nulliparous. Thirty‐seven percent of patients were (non‐Hispanic) Caucasian, 38% (non‐Hispanic) African American, and 22% Hispanic, and less than 3% identified themselves in another race category. There were 280 (56.3%) patients with a BMI of 40.0–44.9, 107 (21.5%) with a BMI of 45.0–49.9, 70 (14.1%) with a BMI of 50.0–59.9, and 40 (8.1%) with a BMI of 60.0 or greater. The median BMI in our selected population was 43.9 (interquartile range: 41.9–48.7, range: 40.0–92.9) (Figure 1). The BMI groups were similar in terms of most demographic variables, including gravida/para, chronic hypertension (cHTN), HDP, gestational diabetes, and race/ethnicity (Table 1). There was an association with increased age and higher BMI, but this finding was not statistically significant (*P =* 0.067) (Table 1).

**FIGURE 1:**
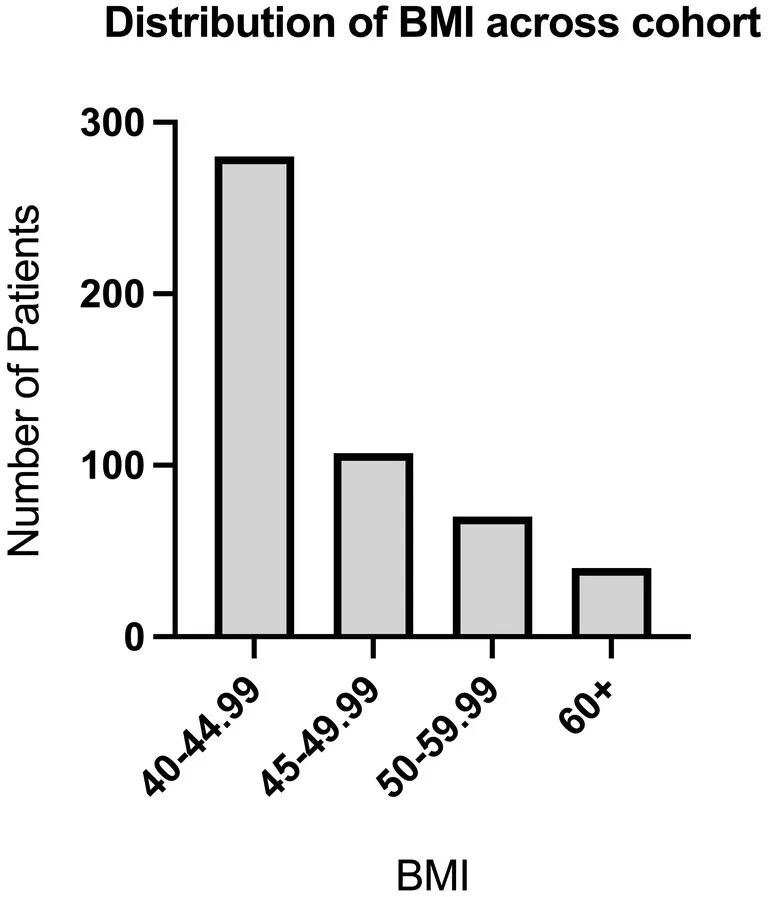
Distribution of body mass index (BMI) across cohort. This graph represents the percentage of patients in each BMI group. Most patients in our study had a body mass index between 40 and 44.9 (56%), with a decreasing percentage of patients in each additional category: BMI 45–49.9 (22%), BMI 50–59.9 (14%), and BMI greater than 60 (8%).

**TABLE 1:** Study population.

	BMI 40–44.9	BMI 45–49.9	BMI 50–59.9	BMI 60+	*P*‐value
Age	27.2 (6.2)	26.9 (6.1)	28.4 (5.5)	29.4 (5.6)	0.067
Gravidity	2.8 (2)	2.7 (2.1)	2.5 (1.7)	2.6 (1.5)	0.780
Nulliparity	141 (51.5)	46 (44.7)	37 (52.9)	23 (57.5)	0.493
Race/ethnicity					0.473
White	95 (34.0)	41 (40.6)	28 (40.0)	16 (40.0)	
Black	106 (39.0)	43 (42.6)	26 (37.1)	9 (22.5)	
Hispanic/Latina	63 (23.2)	15 (14.9)	13 (18.6)	13 (32.5)	
Other	8 (2.9)	2 (2.0)	3 (4.3)	2 (5.0)	

*Note*: Values are presented as *N* (column %) or mean and standard deviation.

Abbreviations: BMI, body mass index.

### 3.2 Labor characteristics

Labor was induced in 73.2% (*n* = 364) of deliveries. Hypertension (either HDP or cHTN) was the indication for 36.2% of IOLs, followed by elective induction (24.9%) and diabetes (either gestational or pre‐existing) (10.2%). A plurality of 7.9% had other indications for IOL, including indications such as non‐reassuring antenatal testing and pre‐labor rupture of membranes. There was a significant difference in IOL indications across BMI groups (*P =* 0.0034). Rates of IOL were significantly different between BMI groups (Table 2, *P =* 0.02733), with the rates of IOL increasing as BMI increased from 40 to 60. The BMI 60+ group had the lowest rates at 62.5%. This finding was also seen with a simple logistic regression analysis that identified an association between IOL and BMI up to a BMI of 60 (Appendix 2).

**TABLE 2:** Labor characteristics.

	BMI 40–44.99	BMI: 45–49.99	BMI: 50–59.99	BMI: 60+	*P*‐value
IOL	198 (70.7)	81 (75.7)	60 (85.7)	25 (62.5)	0.0273*
Indication for IOL					0.0034*
AMA	13 (9.6)	2 (2.3)	1 (1.6)	4 (16)	
Pre‐GDM	1 (0.7)	4 (4.7)	2 (3.3)	0 (0)	
GDM	16 (11.9)	7 (0.81)	5 (8.3)	2 (8)	
cHTN	24 (17.8)	9 (10.5)	20 (33.3)	4 (16)	
HDP	33 (24.4)	22 (25.6)	17 (28.3)	3 (12)	
Oligohydramnios	14 (10.4)	7 (8.1)	3 (5)	2 (8)	
FGR	5 (3.7)	2 (2.3)	2 (0.3)	1 (4)	
Late‐term or post‐dates	10 (7.4)	1 (1.1)	0 (0)	0 (0)	
Other	19 (14.1)	6 (7.0)	2 (3.3)	2 (8)	
Elective	63 (46.7)	21 (24.4)	8 (13.3)	7 (28)	
Labor characteristics					
Internal monitors	148 (52.6)	62 (57.9)	50 (71.4)	18 (0.45)	0.0185*
AROM	184 (65.7)	74 (69.1)	44 (62.9)	28 (70)	0.7891
Meconium	27 (9.6)	11 (10.3)	4 (5.7)	3 (7.5)	0.7105

*Note*: Values are presented as *N* (column %) or mean and standard deviation.

Abbreviations: AMA, advanced maternal age; AROM, artificial rupture of membranes; BMI, body mass index; cHTN, chronic hypertension; FGR, fetal growth restriction; GDM, gestational diabetes; HDP, hypertensive disorder of pregnancy including gestational hypertension; IOL, induction of labor; pre‐GDM, pre‐existing diabetes.

* *P*‐value <0.05.

On multivariate analysis (Table 3), BMI between 50 and 59.99 is found to be an independent predictor of IOL while controlling for other patient characters. Relative to patients with BMI 40–44.99, patients with BMI 50–59.99 have 2.2 times increased odds of IOL (95% confidence interval [CI]: 1.07–4.92, *P =* 0.042). Increasing maternal age, nulliparity, and hypertension (HDP or cHTN) are also found to be independent predictors of IOL. There was also a significant difference across BMI classes in the use of internal monitors (fetal scalp electrode or an intrauterine pressure catheter, *P =* 0.0185; Table 2); the rate of internal monitor use increased from 52.6% to 71.4% as BMI increased from 40 to 60, but the BMI 60+ group had a lower rate of use (45%).

**TABLE 3:** Logistic regression adjusted analyses: Induction of labor.

	Outcome: IOL		
	AOR	95% CI	*P*‐value
BMI			
40*–*44.99	Ref	*—*	*—*
45*–*49.99	1.14	(0.65, 2.01)	0.656
50*–*59.99	2.20	(1.07, 4.92)	0.042*
60+	0.58	(0.28, 1.24)	0.151
Maternal age	1.08	(1.03, 1.13)	0.001*
Nulliparity	2.40	(1.49, 3.93)	<0.001*
HTN	2.85	(1.70, 4.93)	<0.001*
GDM	1.09	(0.52, 2.48)	0.820
GA	0.97	(0.78, 1.22)	0.807

*Note*: Values are presented as adjusted odds ratio (AOR) and 95% confidence interval (CI).

Abbreviations: IOL, induction of labor; BMI, body mass index; Ref, reference group; CI, confidence interval; GA, gestational age; GDM, gestational diabetes mellitus; HTN, hypertension.

* *P*‐value <0.05.

### 3.3 Delivery outcomes

The rates of CD increased with increasing BMI group, but in the 60+ group, the rate of CD was lower; there was not a statistically significant correlation between CD and BMI group (*P =* 0.350) (Table 4). Of all patients who presented in spontaneous labor, 12.7% ultimately underwent a CD. This is in contrast to a 34.6% CD rate in those patients who underwent IOL. There was no significant difference across BMI within the subset of patients with spontaneous delivery (*P =* 0.593). There was a correlation between IOL and CD between BMI groups (*P =* 0.0332).

**TABLE 4:** Logistic regression adjusted analyses: Cesarean section.

	Outcome: Cesarean section		
	AOR	95% CI	*P*‐value
BMI			
40*–*44.99	Ref		
45*–*49.99	1.33	(0.76, 2.28)	0.310
50*–*59.99	1.39	(0.72, 2.68)	0.324
60+	1.30	(0.56, 2.92)	0.527
Maternal age	1.03	(0.99, 1.08)	0.116
Null parity	5.42	(3.27, 9.20)	<0.001*
HTN	1.27	(0.79, 2.04)	0.318
GDM	2.24	(1.15, 4.35)	0.017*
GA	1.16	(0.93, 1.45)	0.182
IOL	2.76	(1.52, 5.27)	0.001*

*Note*: Values are presented as adjusted odds ratio (AOR) and 95% confidence interval (CI).

Abbreviations: BMI, body mass index; Ref, reference group; CI, confidence interval; GA, gestational age; GDM, gestational diabetes mellitus; HTN, hypertension; IOL, induction of labor.

* *P*‐value <0.05.

In patients who had a medically indicated IOL, 37.4% delivered via CD. Only 20.3% of patients who underwent an elective IOL delivered via CD. The relationship between medically indicated induction of labor and CD was statistically significant (*P* = 0.0012). Of those patients who underwent an elective IOL, there was an association with CD by BMI group (*P* = 0.0088). This relationship was not seen among those undergoing a medically indicated IOL (*P* = 0.3588, Table 5). This finding was also seen on a simple logistic regression that identified that BMI did not predict CD among those who underwent an elective IOL versus those who underwent a medically indicated IOL (Figure 2). When reviewing the indication for CD, there were no differences between BMI groups (*P* = 0.5027, Table 5).

**TABLE 5:** Delivery outcomes.

	BMI 40–45.99	BMI 45–49.99	BMI 50–59.99	BMI 60+	*P*‐value
CD	72 (26.3)	34 (34)	24 (34.3)	11 (27.5)	0.350
CD by labor type					
Spontaneous	12 (15.2)	4 (16.7)	0 (0)	1 (6.7)	0.593
Induction	59 (25.8)	32 (37.2)	24 (40.6)	11 (44.0)	0.0332*
Medically indicated	46 (34.1)	23 (38.3)	20 (28.5)	10 (55.6)	0.3588
Elective	13 (14.0)	9 (34.6)	4 (57.1)	1 (14.3)	0.0088*
Indication for cesarean					0.5027
Non‐reassuring FHT	29 (40.3)	12 (35.3)	5 (20.1)	4 (36.4)	
Failed induction of labor	34 (47.2)	19 (55.9)	13 (54.1)	5 (45.4)	
Other	9 (12.5)	3 (8.8)	6 (25)	2 (18.1)	
GA	39.2 (1.0)	39.2 (1.0)	38.6 (0.9)	39.1 (1.3)	<0.001*
BW	3367 (474)	3421 (470)	3225 (442)	3397 (477)	0.050
Shoulder dystocia	7 (2.6)	3 (2.9)	1 (1.5)	0 (0)	0.861
Perineal laceration	3 (1.5)	2 (2.9)	0 (0)	2 (6.9)	0.155
3rd or 4th degree laceration	10 (3.8)	3 (3)	1 (1.5)	2 (5.6)	0.668
Postpartum hemorrhage	22 (8.2)	13 (12.6)	6 (8.6)	4 (10.3)	0.594
APGAR score at 1 min	7.7 (1.6)	7.4 (1.9)	7.6 (1.7)	7.7 (1.4)	0.385
APGAR score at 5 min	8.7 (0.9)	8.6 (1.0)	8.7 (0.8)	8.6 (1.1)	0.611

*Note*: Values presented as *N* (column %), or Mean and standard deviation.

Abbreviations: BMI, body mass index; BW, birth weight reported in grams; CD, cesarean delivery; FHT, fetal heart tones; GA, gestational age.

* *P*‐value <0.05.

**FIGURE 2:**
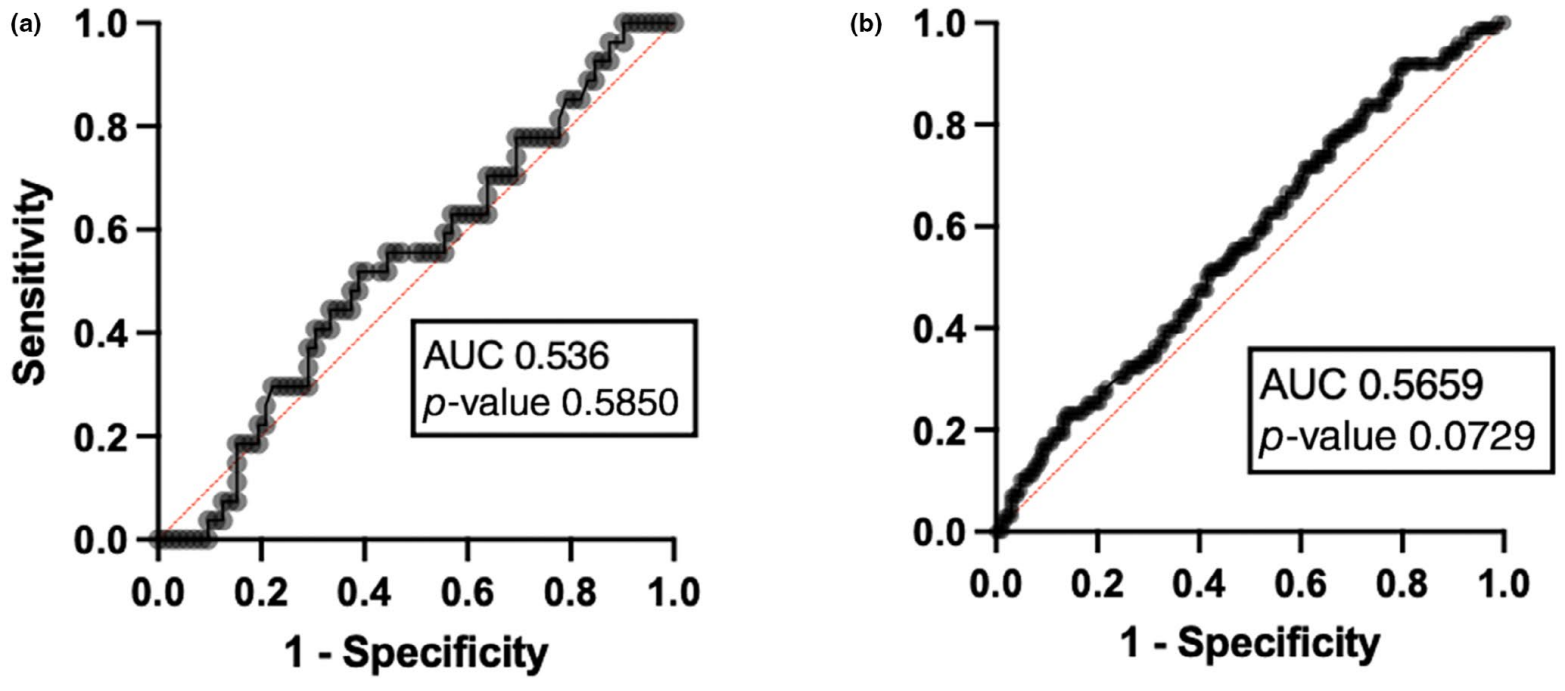
Cesarean delivery by body mass index (BMI) among those that underwent elective versus medically indicated induction of labor (IOL). Receiver operating characteristic (ROC) curves for prediction of cesarean delivery by BMI in patients who underwent elective (a) and medically indicated (b) IOL.

When comparing mode of delivery by IOL versus spontaneous labor, there was a significant association with CD in the BMI 40–44.99 (*P* = 0.0065), BMI 50–59.99 (*P* = 0.0124), and the BMI 60 and greater (*P* = 0.0278) groups (Table 6). This relationship was nearly statistically significant for the BMI 45–49.99 group (0.0502) (Table 6). In multivariate analysis (Table 4), there was no significant difference in the rates of cesarean delivery by BMI group.

**TABLE 6:** Induction of labor and cesarean delivery.

BMI group	CD spontaneous labor	CD IOL	*P*‐value
40*–*44.99	12 (15)	60 (30.9)	0.0065*
45*–*49.99	4 (16)	30 (38.5)	0.0502
50*–*59.99	0 (0)	24 (40)	0.0124*
60+	1 (6.7)	10 (41.7)	0.0278*

*Note*: Values presented as *N* (column %).

Abbreviations: BMI, body mass index; CD, cesarean delivery; IOL, induction of labor.

* *P*‐value <0.05.

Cesarean delivery was strongly associated with nulliparity (adjusted odds ratio 5.42, CI: 3.27–9.20, *P* < 0.001), as well as gestational diabetes (adjusted odds ratio [AOR] = 2.24, CI: 1.15–4.35, *P =* 0.017) and IOL (AOR = 2.76, 1.52–5.27, *P =* 0.001).

Shoulder dystocia occurred in 2.3% of all deliveries, with no difference by BMI group (*P =* 0.863, Table 5). Perineal lacerations (first and second degree) as well as more severe lacerations (third and fourth degree) had a similar representation between groups, and this finding was not statistically significant (*P =* 0.155 and 0.668, respectisvely, Table 5). Postpartum hemorrhage occurred in 9.2% of patients and was similar among groups (Table 5).

### 3.4 Infant outcomes

The average gestational age at delivery was 39.1 ± 1.0 weeks, and BMI between 50 and 59.99 was associated with earlier delivery (*P* < 0.001) (Table 5). The average APGAR scores were 7.6 ± 1.6 at 1 min and 8.7 ± 0.9 at 5 min and showed no difference by BMI (*P =* 0.385 and 0.611) (Table 5).

## 4 DISCUSSION

### 4.1 Main findings

There is a significant association between IOL by BMI class among patients with class III obesity (*P* = 0.0273). Multivariate analysis found that BMI 50.0–59.9 was an independent risk factor for IOL. The rate of IOL ranged from 62.5 to 85.7% among the BMI groups. This is contrast to the IOL rate of 41% at our institution during the study period.

Body mass index was not predictive of CD, but CD was more likely in those patients who presented for induction compared to patients in spontaneous labor. There was a higher likelihood of CD in the medically indicated IOL group (37.4%) compared to the elective IOL group (20.3%) (*P* = 0.0012); at our institution during the study period, the primary cesarean section rate was 26%. Among those who underwent IOL for a medical indication, there was no association between BMI group and CD (*P* = 0.3588), but there was a statistically significant association between elective IOL and BMI group (*P* = 0.0088).

Of note, the BMI 60+ group was found to disagree with the increasing trends that were noted among the other BMI groups. This is likely due to the smaller sample size of this group.

### 4.2 Interpretation considering other evidence

There is disagreement in the literature regarding obese patients who undergo elective IOL. Some evidence shows the odds of CD were lower among patients with obesity who underwent elective induction between 37 and 39 weeks' gestation compared to those who underwent expectant management.^17^ Other studies suggest that patients with obesity who undergo IOL are more likely to have a failed IOL.^18^ Further, women with obesity have a higher rate of CD after late‐term IOL compared to patients with normal BMI.^19^ In our study, the average gestational age was 38.7 ± 1.8 weeks at the time of admission. We found significant relationships between IOL and BMI and between IOL and CD, but this relationship was not preserved when controlling for other factors during the multivariate regression. There was not a direct dose‐dependent relationship between CD and BMI. A possible explanation for the difference in outcomes between studies might be due to evaluating differences among those with class III obesity in our study compared to other studies comparing outcomes in patients with class III obesity against other obesity classes. Studies might also differ because of the variety of indications for labor induction that are not stratified in these analyses as well as the indication for CD. Further studies should control for the indication for induction as well as the indication for CD to determine if increasing maternal BMI is an independent risk factor for failed IOL.

Patients with obesity are at increased risk for late‐term pregnancy and are more likely to undergo induction of labor.^2^, ^3^ Overall, our study confirmed these findings; compared to BMI between 40 and 44.99, patients will BMIs in the 50s have 120% higher odds of labor induction (AOR = 2.20, 95% CI 1.07–4.92 *P =* 0.042). This increased rate was not found in the BMI greater than 60 group. This might be due to the small sample size or the effect size or could perhaps be due to a different etiology. In a study by Kim et al. that evaluated maternal complication rates, including IOL, in patients with BMI of 60 or greater compared to other patients with class III obesity (BMI 40.0–59.9) found that there was a dose‐dependent increased risk of maternal complications as BMI increases.^11^ Interestingly, our study did not find an increased rate of late‐term induction among those with higher BMI; this might be due to IOL occurring due as a result of chronic conditions prior to the late term period. Additional study is needed to better characterize birth outcomes in patients with BMI 60 and greater so we may understand what factors are driving IOL in patients with higher BMI.

Some studies have suggested that pregnant women with BMI >40 have a significantly increased risk of postpartum hemorrhage compared to their non‐obese counterparts.^2^, ^3^ Our study did not identify an increase in hemorrhage as BMI increased. There was no statistically significant difference in postpartum hemorrhage among BMI groups.

The data is mixed on whether obesity is an independent risk factor for shoulder dystocia, and our study found no association between BMI and the incidence of shoulder dystocia.^3^, ^20^ Although some studies have suggested that increasing BMI has a protective effect in preventing third‐ and fourth‐degree lacerations, our study found no differences between groups.^3^, ^21^

Overall, we have found that patients with class III obesity are more likely to require IOL and that those who undergo IOL are more likely to have a CD, but when this relationship was evaluated using multivariate regression, the relationship was not preserved. Further investigation is needed to determine if there are any predictors for unsuccessful IOL in patients with class III obesity. Additionally, research is needed to determine if there is an ideal gestational age for primiparous and multiparous patients with class III obesity to undergo IOL.

### 4.3 Strengths and limitations

The demographics of patients between groups were well matched. The study was limited by its restriction to a single site and the potential for confounding bias. The retrospective study design is inherently limited due to possible inaccurate record‐keeping, interobserver variability, and an inability to control for all confounding factors. Further, data regarding indications for CD such as failure to dilate, failure to descend, or non‐reassuring fetal heart tracing were not captured. Future studies will involve collaboration with additional sites to increase power, and sample size and develop generalizable conclusions for a greater population.

## 5 CONCLUSION

In patients with a BMI between 50.0 and 59.9, there is an increased rate of IOL but no associated increase in CD rate. The literature states that elective IOL will not increase a patient's risk of CD, but our investigation of patients with class III obesity coincides with these conclusions. Our study suggests that as BMI increases, there is an increased risk of CD with elective IOL as BMI increases. Further, our study found that those undergoing a medically indicated IOL are more likely to undergo CD compared to those undergoing an elective IOL. Future studies are needed to better characterize outcomes for patients with a BMI of 60 or greater. Providers need inclusive data to best counsel patients with obesity on delivery planning.

## AUTHOR CONTRIBUTIONS

ER: writing manuscript, editing, formatting figures, study design. BM: conceptualization, study design, data collection. JG: data‐analysis, supervision, AM: data‐analysis, RP manuscript editing, study design, EM supervision, conceptualization, JK supervision, manuscript editing, project conception.

## FUNDING INFORMATION

The authors have no funding to report for the development of this work.

## CONFLICT OF INTEREST STATEMENT

The authors have no conflicts to report.

## ETHICS STATEMENT

IRB approval was obtained from the University of Louisville Internal Review Board. Approval was granted February 2020 prior to beginning this study.

## Supporting information


Appendix S1.
**Appendix S2.** Induction of Labor by BMI.

## Data Availability

Research data are not shared.
