# Supplementary material for: Rate of cesarean delivery among patients with class III obesity at term based on body mass index: A retrospective cohort study
Source: Int J Gynaecol Obstet. 2026 May 11;175(2):741–8. doi: 10.1002/ijgo.71060 (PMC13674836; doi:10.1002/ijgo.71060)
Supplement: Supplementary file 1 [file IJGO-175-741-s001.docx]

Appendix 1.

| Outcome | Definition |
| --- | --- |
| Age | Age at time of delivery |
| Gravidity | Number of times the patient has been pregnant |
| Nulliparity | First pregnancy |
| DM in Pregnancy | A diagnosis of either gestational or pre-existing or pre-gestational diabetes |
| HDP | A diagnosis of hypertension during pregnancy including chronic hypertension, gestational hypertension, or preeclampsia |
| Race/Ethnicity | Patient reported race or ethnicity at time of admission to labor and delivery |
| Induction of Labor | If the patient underwent induction of labor on admission to labor and delivery |
| Indication for IOL | The History and Physical at time of admission was reviewed and the indication for induction was listed. |
| Internal Monitors | The patient’s labor course was reviewed and if a fetal scalp electrode or intrauterine pressure catheter was utilized this was considered positive. |
| Spontaneous Rupture of Membranes | The patient’s labor course was reviewed and if spontaneous rupture of membranes occurred this was considered positive |
| Meconium | The patient’s labor course was reviewed and if meconium fluid was noted, this was considered positive. |
| Cesarean Delivery | Delivery note was reviewed and those who had a cesarean delivery were considered positive |
| CD By Labor Type | The patient’s chart was reviewed and it was noted whether the patient presented in spontaneous labor or for an induction |
| Gestational age | The delivery note was reviewed and the gestational age at time of delivery was noted. |
| Birth weight | The delivery note was reviewed, and the birth weight was recorded in grams. |
| Shoulder dystocia | The delivery note was reviewed and those with a complication of shoulder dystocia were included |
| Perineal Laceration | The delivery note was reviewed and those with a perineal laceration reported as a first, second, third or fourth degree laceration based on ACOG criteria. |
| 3rd or 4th degree laceration | The delivery note was reviewed and those with a third or fourth degree laceration per ACOG criteria were included |
| EBL | The delivery note was reviewed and the volume of blood at the time of delivery was recorded |
| Postpartum hemorrhage | Using chart review the total EBL was calculated and those with volume greater than 1000cc were considered positive |
| Apgar at 1min | The delivery note was reviewed and the neonates Apgar score at 1 minute of life was recorded. |
| Apgar at 5min | The delivery note was reviewed and the neonates Apgar score at 5 minutes of life was recorded. |

Appendix 2: Induction of Labor by BMI

Induction of Labor (IOL); Body Mass Index (BMI) Receiver operating characteristic (ROC)

ROC curves for prediction of IOL by BMI. Due to the small sample size in the BMI 60 and greater group, the data was analyzed only including those with a BMI 40-59.99.

P value <0.05 *
